# Reflexões sobre a profilaxia dos arbovírus na América Latina

**DOI:** 10.26633/RPSP.2019.81

**Published:** 2019-09-30

**Authors:** Eduardo Dias Wermelinger

**Affiliations:** 1 Fundação Oswaldo Cruz (FIOCRUZ) Escola Nacional de Saúde Pública Rio de Janeiro (RJ) Brasil Fundação Oswaldo Cruz (FIOCRUZ), Escola Nacional de Saúde Pública, Rio de Janeiro (RJ), Brasil.

Ao Editor:

O recém-publicado panorama da dengue nos estados membros do Mercosul (1) aborda um problema de elevada importância para a saúde pública na América Latina, mas que merece ser melhor discutido para orientar as ações governamentais. Inicialmente, é pertinente observar que o atual cenário epidemiológico da dengue, em particular no Brasil, está acompanhado pela disseminação de outros arbovírus também transmitidos pelos mosquitos urbanos (com destaque para *Aedes aegypti*), como Zika, Chikungunya, febre amarela e, mais recentemente, Mayaro. O incremento recente, na presente década, na circulação desses múltiplos arbovírus na América Latina mostra uma realidade epidemiológica complexa e preocupante, que não é mais exclusiva da dengue.

É preciso ponderar a recomendação do estudo de que os países associados ou membros do Mercosul, principalmente o Brasil, precisam identificar os fatores que favorecem o aumento da propagação da endemia (p. 1). Existe na literatura uma boa compreensão dos determinantes sociais, ambientais e climáticos que favorecem a proliferação dessas arboviroses no meio urbano. Urbanização descontrolada, elevadas densidades demográficas (favelas), altas temperaturas associadas às chuvas, saneamento precário, exclusão social e, no Brasil em particular, a violência, são determinantes que favorecem a proliferação dos mosquitos e a circulação dos arbovírus e dificultam ou inviabilizam a eficácia dos serviços de controle de vetores e da vigilância epidemiológica. Portanto, o desafio não é identificar os fatores ou determinantes que favorecem o aumento da propagação; é, isso sim, identificar formas de suplantar o conjunto desses determinantes para alcançar efetivos resultados profiláticos. O estudo recomenda ainda a necessidade urgente de fortalecer as ações intersetoriais entre Estados do Mercosul. No entanto, na prática, essa recomendação não contribui para contornar os determinantes em questão e alcançar uma eficaz profilaxia das arboviroses nas áreas endêmicas urbanas.

Na ausência de vacinas para o conjunto dos arbovírus circulantes, a profilaxia depende principalmente do controle dos mosquitos vetores. O histórico de epidemias e a introdução dos novos vírus revela que as atuais diretrizes usadas no controle de vetores não oferecem a necessária eficiência. Portanto, colocando de lado a evidente necessidade de desenvolver vacinas, para fins profiláticos imediatos o verdadeiro e urgente desafio é buscar estratégias eficazes para o controle dos mosquitos vetores.

Diante desse desafio, novas tecnologias vêm sendo pesquisadas e testadas. No Brasil e em vários países ao redor do mundo, investimentos e esforços vêm sendo feitos na liberação de mosquitos geneticamente modificados (por exemplo, machos estéreis) ou infectados com a *Wolbachia*. No entanto, a despeito dos conflitos éticos e metodológicos dessas tecnologias (2), ainda não há evidências da eficácia profilática dessas liberações, sobretudo em amplas áreas endêmicas tropicais.

Do ponto de vista teórico-metodológico, é muito relevante e desejável que as estratégias de controle de vetores priorizem as ações de manejo ambiental, que objetivam a eliminação duradoura ou permanente dos criadouros, reduzindo a dependência dos paliativos inseticidas. O manejo ambiental é incontestavelmente o método de controle de vetores mais eficaz, econômico e sustentável (duradouro). As tradicionais políticas de controle vetorial apostam na mobilização comunitária para eliminar os criadouros urbanos, em geral através do uso dos meios de comunicação. Entretanto, apesar da ampla literatura sobre o envolvimento comunitário na eliminação dos criadouros, não há evidências de que esse envolvimento seja viável de forma suficiente, com efetivos resultados profiláticos em grandes centros urbanos.

O histórico de insucesso das estratégias de controle de vetores em bloquear a circulação dos vírus da dengue e demais arbovírus e a pertinência em priorizar as ações de manejo ambiental devem nortear o desafio delineado acima, de elaborar novas estratégias eficazes de controle dos vetores. Nesse desafio, é importante fazer uma revisão crítica dos atuais conceitos, crenças e metodologias utilizadas na vigilância e no controle vetorial. É conveniente refletir sobre a pertinência de usar novas habilidades e expertises nos serviços de controle de vetores, de forma a contornar os obstáculos sociais e ambientais que têm impedido a eficácia das ações de controle. Por exemplo, pode ser interessante envolver assistentes sociais e urbanistas nas equipes de controle de vetores. De forma análoga, é interessante pensar em mecanismos legais (leis) inteligentes que incentivem a participação comunitária nas ações de manejo ambiental para a eliminação dos criadouros. O objetivo dessas reflexões é contribuir para a busca de ações eficazes para a profilaxia das arboviroses nas Américas.

## Declaração.

As opiniões expressas no manuscrito são de responsabilidade exclusiva dos autores e não refletem necessariamente a opinião ou política da RPSP/PAJPH ou da Organização Pan-Americana da Saúde (OPAS).
